# The Hospital World

**Published:** 1911-04-08

**Authors:** 


### The Hospital World.

### ELECTIONS AND APPOINTMENTS.

Lord Beauchamp, First Commissioner of Works, has been elected president of the Royal Institute of Public Health.

Dr. Eric E. Young, M.R.C.S., L.R.C.P., M.B., has been appointed honorary surgeon, and Dr. S. McMurray, M.B., B.Ch., F.R.C.S., ophthalmic surgeon, to the Longton Cottage Hospital, Staffs.

Lord Donoughmore has accepted the treasurership of the London Homœopathic Hospital, Great Ormond Street, vacant by the death of Lord Cawdor.

The following are the new members of the Committee of Management of Queen Charlotte's Lying-in Hospital: Viscount de Vesci, Sir Maurice Fitzgerald, Bart., C.V.O., Dr. William J. Gow, F.R.C.P., Mr. W. Raymond Greene, M.P., Mr. Henry D. Harben, Mr. L. H. Shore Nightingale, Mr. Arthur H. Pott, Mr. Charles T. Pulley, Mr. Howard J. Walford. Sir Samuel Scott, Bart., M.P., was re-elected chairman and Mr. Frederick W. Hunt vice-chairman.

The Council of Sheffield University has made the following appointments: Mr. R. J. Pye-Smith, Ch.M. Sheffield, F.R.C.S., as Emeritus Professor of Surgery; Mr. Arthur K. Connell, F.R.C.S. Edin., to the lectureship in surgery rendered vacant through Dr. Sinclair White's appointment to the professorship of surgery; Mr. J. D. Fiddes, M.A., B.Sc., M.B., Ch.B. Aberd., to the demonstratorship in anatomy, in succession to Mr. Alexander Wilson, resigned.

Sir Edward Wood has been unanimously elected chairman, and Sir Arthur Grey Hazlerigg, Bart., deputy-chairman of the Board of Governors of the Leicester Infirmary for the ensuing year. In thanking the Board for electing him chairman for the tenth time, Sir Edward Wood added that there was still a great work to be done which he thought he had more knowledge of at present than any other member of the Board. He hoped to suggest ways and means for increasing the income. Important administrative matters were also under consideration. Mr. S. Fielding Johnson was elected chairman of the House Committee.

The Marquis of Winchester has been re-elected president of the Royal South Hants and Southampton Hospital. Mr. R. S. Hankinson, hon. treasurer; and, as members of the committee, the Rev. C. H. Compton, Capt. R. W. Hope, R.N., Sir James Lemon, Mr. P. Pepper, and Mr. P. Leckie were also re-elected.

### PERSONALIA.

Mr. G. Q. Roberts, M.A., has accepted the Hon. Secretaryship of the fund now being raised for a memorial to Miss Nightingale.

### Resignations at the Royal Surrey.

Mr. H. A. Powell has resigned his appointment as honorary treasurer to the Royal Surrey County Hospital, and, as coincidence will have it, is to direct the local education authorities as chairman of the Surrey Education Committee. A few years ago the fitness of Mr. Powell for his new position could not have been judged from his previous experience. To-day the advantages of a hospital treasurer becoming an important educational official need no emphasis. The honorary secretary, Mr. E. Gibson, has resigned also, and thus the Surrey Hospital is to see two very important changes in its institutional staff. The vacant posts are to be filled by two Army officers, Lieut.-General Sir Edmund Elles having been elected hon. treasurer and Major C. J. Matson honorary secretary. We shall be glad to hear of the hospital's progress as any new developments occur.

### The late Mr. Corcoran, of Loughborough.

By the death of Dr. Thomas Francis Corcoran, of Loughborough, at the age of fifty-eight years, whilst at Nazareth, Palestine, the Town Council of Lougborough loses its Medical Officer of Health, and the Loughborough Hospital and Dispensary its Hon. Consulting Physician. Mr. T. F. Corcoran was awarded the diploma of the R.C.S., Ireland, in 1880, and of the Royal Apothecaries' Hall, Dublin, in 1882. He was senior medical officer of the Loughborough Medical Aid Association, and was for a long period an active member of the Town Council of Loughborough. At the time of his death he was Medical Officer of Health for the Loughborough and Leake District Councils, and a permanent director of the Loughborough Permanent Building Society.

### An M.O.H. Pioneer.

A week ago to-day Dr. Robert Trimble brought to a close by final resignation a connection of thirty years with the Preston Infirmary, of which he has been honorary medical officer. His retirement is due to family reasons, and will take the form of a country practice at Burgh-by-Sands, a Cumberland village. But Dr. Trimble's position at the infirmary has not been by any means his only link with the district, for as Medical Officer of Health he has had charge of the public health of Walton-le-Dale for thirty-four years. Under Dr. Trimble Walton has developed from a drainless, ill-lighted suburb into a great urban district, properly paved, lit, and with a modern sewerage system, and a pure supply of water. Of Scotch descent and with an Irish upbringing, Dr. Trimble completed his medical education at Dublin, where he succeeded his father in many offices of the public health, such as it was in those days, and at Glasgow, where he obtained his M.D. It is popularly believed that his long residence at Walton was the result of a chance liking for the neighbourhood, which he experienced during a visit many years ago. It has been said of him that he originated local government at Walton. The Preston Infirmary owes many services to him.

